# Fecal filtrate transplantation protects against necrotizing enterocolitis

**DOI:** 10.1038/s41396-021-01107-5

**Published:** 2021-09-22

**Authors:** Anders Brunse, Ling Deng, Xiaoyu Pan, Yan Hui, Josué L. Castro-Mejía, Witold Kot, Duc Ninh Nguyen, Jan Bojsen-Møller Secher, Dennis Sandris Nielsen, Thomas Thymann

**Affiliations:** 1 Department of Veterinary and Animal Sciences, Faculty of Health and Medical Sciences, University of Copenhagen, Copenhagen, Denmark; 2 Department of Food Science, Faculty of Science, University of Copenhagen, Copenhagen, Denmark; 3 Department of Plant and Environmental Sciences, University of Copenhagen, Copenhagen, Denmark; 4 Department of Veterinary Clinical Sciences, Faculty of Health and Medical Sciences, University of Copenhagen, Copenhagen, Denmark

**Keywords:** Bacteriophages, Microbial ecology, Inflammatory bowel disease

## Abstract

Necrotizing enterocolitis (NEC) is a life-threatening gastrointestinal disorder afflicting preterm infants, which is currently unpreventable. Fecal microbiota transplantation (FMT) is a promising preventive therapy, but the transfer of pathogenic microbes or toxic compounds raise concern. Removal of bacteria from donor feces by micropore filtering may reduce this risk of bacterial infection, while residual bacteriophages could maintain the NEC-preventive effects. We aimed to assess preclinical efficacy and safety of fecal filtrate transplantation (FFT). Using fecal material from healthy suckling piglets, we compared rectal FMT administration (FMT, *n* = 16) with cognate FFT by either rectal (FFTr, *n* = 14) or oro-gastric administration (FFTo, *n* = 13) and saline (CON, *n* = 16) in preterm, cesarean-delivered piglets as models for preterm infants. We assessed gut pathology and analyzed mucosal and luminal bacterial and viral composition using 16S rRNA gene amplicon and meta-virome sequencing. Finally, we used isolated ileal mucosa, coupled with RNA-Seq, to gauge the host response to the different treatments. Oro-gastric FFT completely prevented NEC, which was confirmed by microscopy, whereas FMT did not perform better than control. Oro-gastric FFT increased viral diversity and reduced Proteobacteria relative abundance in the ileal mucosa relative to control. An induction of mucosal immunity was observed in response to FMT but not FFT. As preterm infants are extremely vulnerable to infections, rational NEC-preventive strategies need incontestable safety profiles. We show in a clinically relevant animal model that FFT, as opposed to FMT, efficiently prevents NEC without any recognizable side effects.

## Introduction

Gut colonization after birth is essential for development of the host immune system. Yet, it has become increasingly clear that microbial perturbation resulting in deviation from the normal gut microbiota developmental trajectory is a risk factor and possibly a contributing factor for a range of neonatal diseases. Gut dysbiosis is of particular concern for preterm infants due to their impaired microbial host defense and high susceptibility to life-threatening infections [1].

Necrotizing enterocolitis (NEC), a lethal inflammatory and necrotic bowel disease mainly affecting very preterm infants, is a prominent example of a disease that is tightly coupled with gut dysbiosis [2]. Major differences between studies of the gut microbiome in preterm infants [3] complicate the elucidation of a clear NEC-associated microbiota, but increased Proteobacteria and reduced Bacteroidetes relative abundances are unifying features of a prediagnostic NEC microbiota across neonatal units [4]. At single hospital sites, the microbiota preceding NEC diagnosis is usually characterized by reduced bacterial diversity, lack of obligate anaerobes and increased relative abundance of single facultative anaerobes often belonging to the family of Enterobacteriaceae [5–7].

In the search for better bacterial therapies, we recently showed a proof of principle for fecal microbiota transplantation (FMT) against NEC, using cesarean-delivered preterm pigs as recipients and breastfed, term pigs as donors [8]. Whereas the beneficial effect of FMT on gut pathology was unequivocal, and there was a significant engraftment of perceived beneficial donor bacteria into the recipients, the safety profile raised some concern, as indicated by increased sepsis incidence and mortality, when FMT was administered orally. Besides bacteria, the fecal matrix consists of archaea, eukarya, viruses, microbial secretome and metabolome, any of which may be attributable to the benefits and adversities of FMT. Accordingly, any means to reduce the complexity of the donor fecal matrix, while maintaining its therapeutic effects is an advancement towards developing a clinically feasible therapy against NEC.

Bacteriophages (referred to as phages in the following) are viruses infecting bacteria in a host-specific fashion, and are omnipresent across all bacteria-containing ecosystems including the mammalian gut. During early gut colonization, phages and bacteria dynamically interact and influence each other’s composition [9, 10]. Interestingly, a small case series of *Clostridioides difficile* infection patients receiving sterile donor fecal filtrate transplantation (FFT) reported cure rates equivalent to regular FMT treatment and attributed the effect to donor phages [11]. In mice, FFT from lean donors to recipients on a high fat diet reduces weight gain and protects against metabolic syndrome development [12]. Accordingly, phages might mediate the beneficial effect of FMT on NEC.

We hypothesized that FFT would be safe and equally effective as FMT for NEC prevention. Using cesarean-delivered preterm pigs as models for very preterm infants, we compared the clinical and gut microbiological effects of FFT by different routes of administration with FMT and control. In the following, we present data that demonstrates superior efficacy of FFT relative to FMT as well as an inconspicuous safety profile. We show that the mucosal microbiome of FFT recipients is enriched in phages and depleted of NEC-associated bacteria, and that this unlike FMT is achieved without significant induction of host mucosal immunity. FFT is a completely novel preventive modality with the potential to drastically reduce the burden of NEC.

## Materials and methods

### Animal experimental procedures

**Fig. 1:**
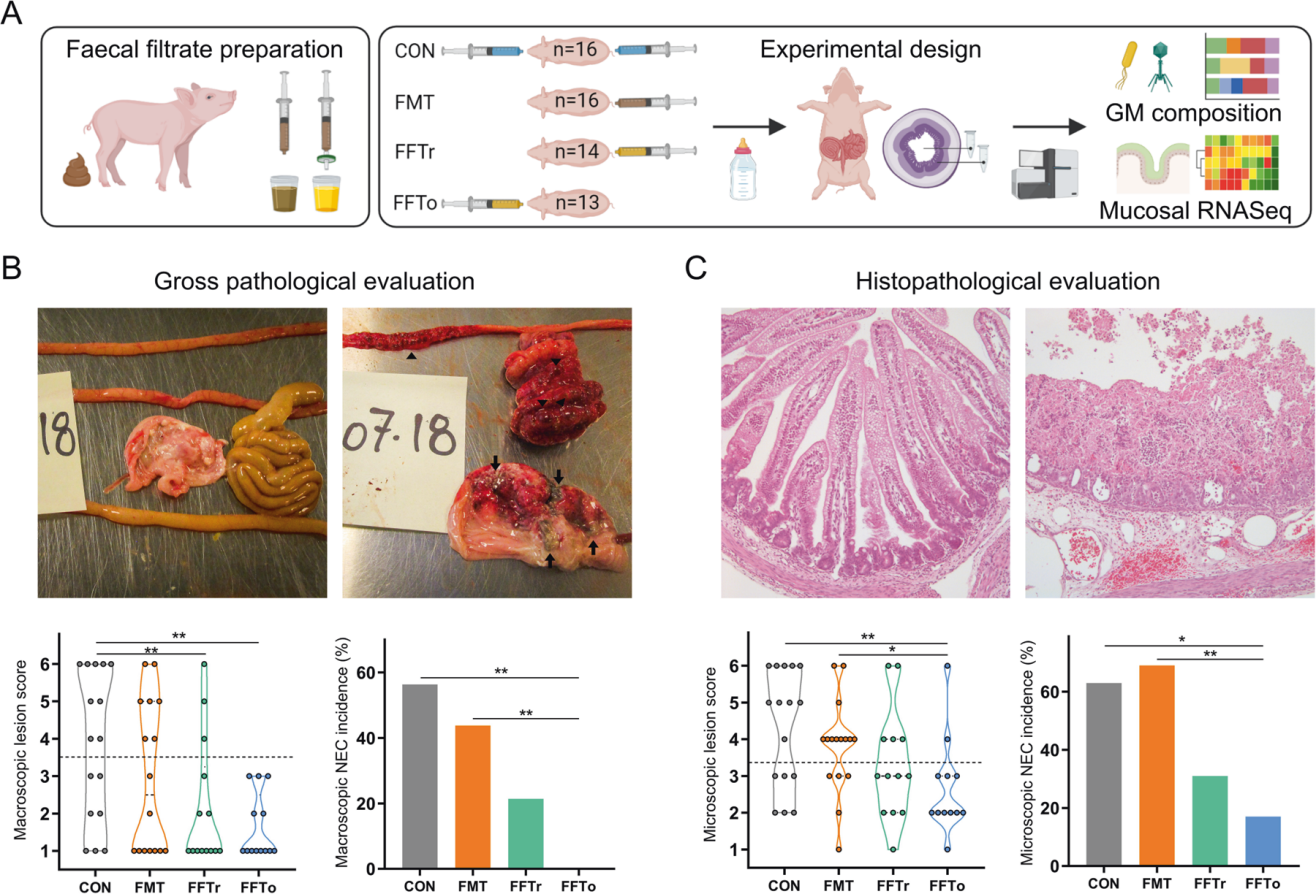
Gut pathological evaluation. **A** Graphical presentation of the study design. **B** Representative necropsy photographs of pig stomach, small intestine and colon with minimal (upper left) or severe pathological changes (upper right). Arrows point to necrotic patches in the mucosa, and arrowheads highlight macroscopic pneumatosis intestinalis. Pathological severity (lower left) and macroscopic NEC incidence (lower right) are shown. **C** Representative micrographs of hematoxylin & eosin stained intact (upper left) and severely disrupted small intestine (upper right) captured at ×10 magnification. Histopathological severity (lower left) and microscopic NEC incidence (lower right) are showed. Asterisk and double asterisks denote statistical probability levels below 0.05 or 0.01, respectively.

The Danish Animal Experiments Inspectorate approved all experimental procedures (license no. 2014-15-0201-00418). Seventy-five conventional crossbred piglets (Landrace x Yorkshire x Duroc) from three healthy sows were delivered by cesarean section at 90% gestation. Birth, resuscitation and housing conditions are described in detail elsewhere [8]. We stratified the animals by sex and birth weight, and randomly allocated them to four groups (Fig. 1A) receiving fecal microbiota transplantation rectally (FMT), fecal filtrate transfer rectally (FFTr), fecal filtrate transfer oro-gastrically (FFTo), or saline as control (CON). All animals received increasing volumes of infant formula by tube feeding (24–96 ml/kg/d, composition in Supplementary Table S1) while simultaneously decreasing parenteral nutrition supplement (96–48 ml/kg/d, Kabiven, Fresenius Kabi, Copenhagen, Denmark).

### Fecal microbiota and filtrate transplantation

Colon luminal content was collected from five 10-day-old piglets and then pooled, gently homogenized and frozen in 10% sterile glycerol. For the FMT solution, thawed fecal material was diluted in sterile saline to 0.05 g/ml and filtered through a 70-µm cell strainer. The FFT solution was prepared in advance as previously described [13]. Briefly, thawed fecal material was diluted to the same concentration as above, homogenized, centrifuged at 5000 × *g* for 30 min at 4 °C, and supernatant filtered through a 0.45 µm PES filter (Minisart^®^ High Flow Syringe Filter, Sartorius, Göttingen, Germany). The purity and virus-like particle concentration was assessed by SYBR gold staining and epifluorescence microscopy, where bacterial cells are easily distinguished by size and fluorescence intensity [14]. Twice daily on days 1 and 2 after birth, animals received 0.5 ml treatment solution [8]. Rectal administration (CON, FMT, FFTr) was performed with a soft rubber probe placed 3–5 cm into the rectum, whereas oro-gastric solutions (CON, FFTo) were administered in the feeding tube followed by flushing with 1 ml of sterile water.

### Clinical monitoring and euthanasia

Animals were monitored by experienced personnel, and daily weights and stool patterns recorded. Animals presenting with clinical signs of NEC or systemic illness throughout the experiment were immediately euthanized. On day 5, animals were deeply anaesthetized and euthanized with a cardiac injection of barbiturate. Intestinal permeability was assessed by urinary lactulose-to-mannitol ratio as previously described [15]. Abdominal organs were excised and weighed, and gross pathological changes of the stomach, small intestine, and colon were assessed in accordance with an established six-grade NEC scoring system by a pathologist blinded to the investigation [8]. The highest grade assigned expressed the disease severity, and NEC diagnosis was defined as pathology grade 4 (extensive hemorrhage) or above. Luminal content was collected from the ascending colon for gut microbiota analysis. Three biopsies were collected along the small intestine for lactase activity measurement [15]. Biopsies of distal ileum and ascending colon were fixed in paraformaldehyde and later embedded in paraffin, sectioned and stained with hematoxylin and eosin for histopathological evaluation, where microscopic NEC was defined as histopathology grade 4 or above (Supplementary Fig. S1). Finally, a 10 cm section of distal ileum was inverted, washed in sterile saline and blotted to remove residual fluid. Mucosa tissue was then scraped off using sterile object glass and cryopreserved for gut microbiota analysis and host transcriptomic analysis.

### 16S rRNA gene amplicon sequencing

The bacterial compositions of distal ileal mucosa and gut luminal content were determined by 16S rRNA gene (V3-region) amplicon sequencing on a NextSeq using v2 MID, 300-cycle, paired-end chemistry (Illumina, San Diego, CA, USA). Total DNA was extracted using Bead-Beat Micro AX Gravity Kit (A&A Biotechnology, Gdynia, Poland) according to the manufacturer’s instructions. Library preparation followed a previously published protocol [16]. The average amplicon sequencing depth was 35,840 reads per sample (min. 9286 and max. 63,584 reads). For a detailed description of the 16S rRNA gene amplicon sequencing bioinformatics workflow, see Supplementary Methods.

### Virome sequencing

The viral content from the same gut mucosa and luminal samples were purified, DNA extracted and library constructed, sequenced and analyzed as previously described [12, 13]. The average sequencing depth for the viral metagenome was 6,158,777 reads per sample (min. 72,077 and max. 13,788,165 reads). The virome sequencing bioinformatics workflow is presented in details in Supplementary Methods.

### RNA-Seq

Global transcriptomic patterns were investigated in RNA extracts from ileal mucosa by RNA-Seq approach. Total RNA was isolated with RNeasy Micro Kit (Qiagen), and 1.5 μg RNA per sample was used for library construction. Sequencing libraries were constructed using NEBNext Ultra RNA library Prep Kit for Illumina (New England Biolabs, Ipswich, MA, USA) and sequenced on the Illumina HiSeq 4000 platform (Illumina) with paired-end 150-bp reads production. The RNA-Seq bioinformatics details are available in Supplementary Methods.

### Systemic immune cell characterization

Complete blood cell counts and basic T cell phenotyping was performed in blood samples collected on days 3 and 5, as previously described [17].

### Statistics

NEC scores and urinary lactulose-mannitol ratio were analyzed by Kruskal–Wallis tests. NEC incidence, rectal bleeding and growth failure incidences were analyzed by Fisher’s exact tests. Weighted Lactase activity across three small intestinal segments was analyzed by two-way ANOVA, and remaining continuous data were analyzed by one-way ANOVA. Probability levels below 0.05 were considered significant.

## Results

### Initial clinical course

Among the 75 cesarean-delivered preterm piglets, nine were excluded before randomization (e.g. failed resuscitation, stillbirth), whereas the remaining 66 animals were group allocated. An additional seven animals were euthanized preschedule for reasons not related to the interventions (respiratory failure, iatrogenic complications). Two animals were euthanized preschedule with clinical NEC signs (1 CON, 1 FFTr), whereas the remaining 57 animals survived until day 5. During the course of the experiment, we observed rectal bleeding in 31% (5/16) of CON and 19% (3/16) of FMT animals relative to 0% (0/13) in both FFT groups (*p* *<* 0.05 vs. CON).

### Gut pathological evaluation

We performed gross examination of the gastrointestinal tract supported by histopathological assessment of ileum and colon to evaluate the severity and extent of NEC-like lesions. The NEC-like pathological phenotype in formula fed preterm pigs consisted of extensive hemorrhage with or without patchy necrosis of the mucosa and pneumatosis intestinalis, mostly affecting the ascending and transverse colon, and to a lesser extent ileum and stomach (Fig. 1B). At the microscopic level, the pathological observations included subtle changes to the mucosal architecture, progressing from epithelial sloughing and hemorrhage to complete destruction of mucosal integrity among the most severe cases (Fig. 1C).

In this setting, oro-gastric FFT administration markedly reduced macroscopic NEC severity (*p* *<* 0.01, Fig. 1B) and consequently reduced NEC incidence to 0% (*p* *<* 0.01 vs. CON). Rectally administered FFT also reduced NEC severity (*p* *<* 0.01) but not incidence relative to CON. However, rectally administered FMT, which we previously found to be clearly NEC protective [8], failed to reduce NEC severity and incidence relative to CON. No bleeding occurred after rectal fluid administration, and no rectal lesions were observed at necropsy in rectally administered animals. The microscopic evaluation supported the macroscopic effects of oro-gastric FFT, which reduced the histopathological NEC severity and incidence relative to both CON and FMT (all *p* *>* 0.05, Fig. 1C), whereas no significant effects were found for rectally administered FFT on microscopic level.

### Safety assessment

**Fig. 2:**
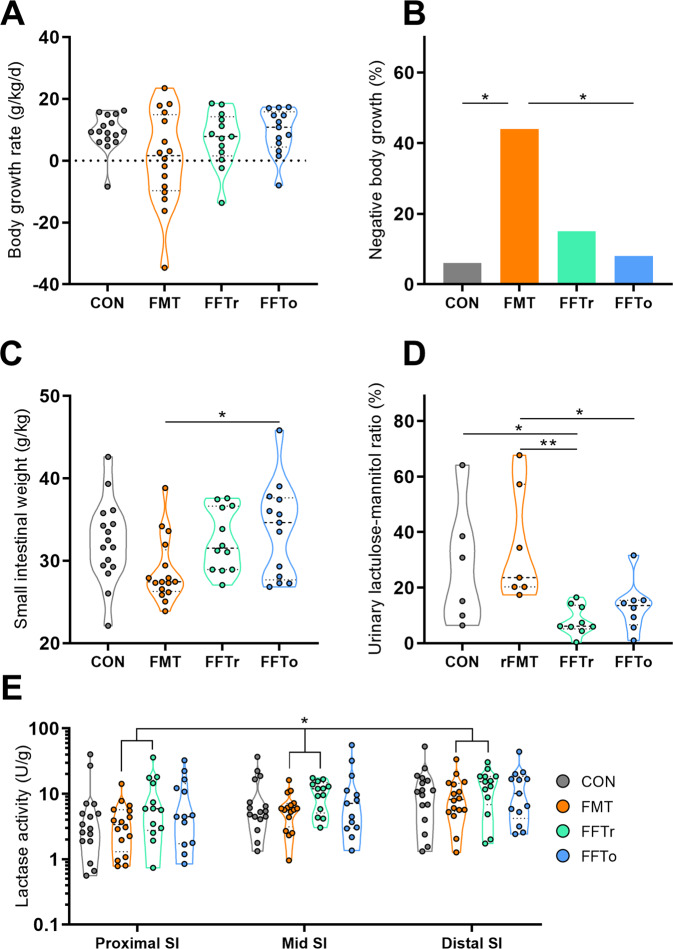
Safety assessment. **A** Relative body growth rate from birth to day 5. **B** Proportion of animals with negative body growth rate from birth to day 5. **C** Relative small intestinal weight. **D** Small intestinal permeability. **E** Brush-border lactase enzyme activity in three segments of small intestine. SI small intestine; Asterisk and double asterisks denote statistical probability levels below 0.05 or 0.01, respectively.

We next investigated a series of safety parameters. Initially, we found that the FMT group had a significantly higher proportion of animals with a negative body growth rate compared with CON and FFTo (both *p* *<* 0.05, Fig. 2A, B). Furthermore, the relative weight of the small intestine but not the colon was lower in FMT animals relative to FFTo (*p* *<* 0.05, Fig. 2C). Additionally, the urinary lactulose-mannitol ratio, an in vivo marker of small intestinal permeability, was robustly decreased by FFTr (*p* *<* 0.05 vs. CON, Fig. 2D), whereas the FMT group had a significantly higher permeability than both FFT groups (both *p* *<* 0.05). Finally, the lactase enzymatic activity, a measure of mucosal integrity, was significantly decreased in the FMT group relative to FFTr (Fig. 2E).

### Bacterial composition of gut mucosa and lumen

**Fig. 3:**
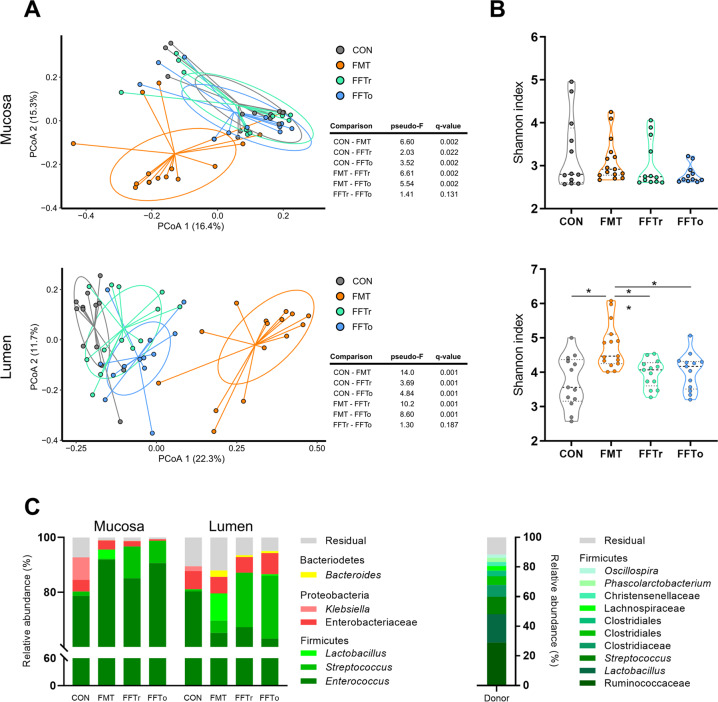
Bacterial composition of gut mucosa and lumen. **A** Principal component analysis plots visualizing beta diversity based on unweighted UniFrac metrics. FDR-adjusted probability levels of pairwise comparisons are reported in adjacent tables. **B** Shannon index as a measure of alpha diversity. **C** Relative bacterial abundance summarized at genus level. Genera with more than 1% relative abundance across groups were included. Bacterial composition of donor fecal material is included as reference. Asterisk and double asterisks denote statistical probability levels below 0.05 or 0.01, respectively.

In general, the composition of mucosa-associated bacteria differed from luminal bacteria (*R*^2^ = 0.11, *p* *<* 0.001, Supplementary Fig. S2). Intervention effects were seen in bacterial composition of both the mucosa (*R*^2^ = 0.17, *p* *<* 0.001, Fig. 3A) and luminal compartments (*R*^2^ = 0.27, *p* *<* 0.001). The greatest effect was observed in the FMT group, but both FFT groups were also significantly different from CON in both mucosa and gut lumen. However, FFT route of administration (FFTr vs FFTo) did not affect the bacterial composition. The Shannon index of mucosa-associated bacteria was similar among groups, but interestingly a positive correlation between Shannon index and small intestinal NEC severity was observed (*p* *<* 0.001, Supplementary Fig. S3). The luminal bacterial Shannon index increased only in response to FMT treatment (Fig. 3B).

Relative to CON, most relative differentially abundant bacterial OTUs were observed in the luminal compartment of FMT animals, whereas the number of differentially abundant mucosa-associated bacteria were comparable between treatments (Supplementary Fig. S4). FFT treatment substantially increased *Streptococcus* relative abundance but not *Lactobacillus* relative to CON, whereas FMT conversely increased *Lactobacillus* relative abundance but not *Streptococcus*, although the donor stool contained high proportions of both these genera (Fig. 3C). Interestingly, while the relative abundance of Proteobacteria (~10%) was similar among groups in the gut lumen, <1% Proteobacteria were detected from the mucosa of oro-gastrically administered FFT animals, whereas ~10% of the mucosal microbiota of CON consisted of Proteobacteria (e.g. Enterobacteriaceae, *Klebsiella*) and ~5% for FMT (Fig. 3C).

### Viral and phage composition of gut mucosa and lumen

**Fig. 4:**
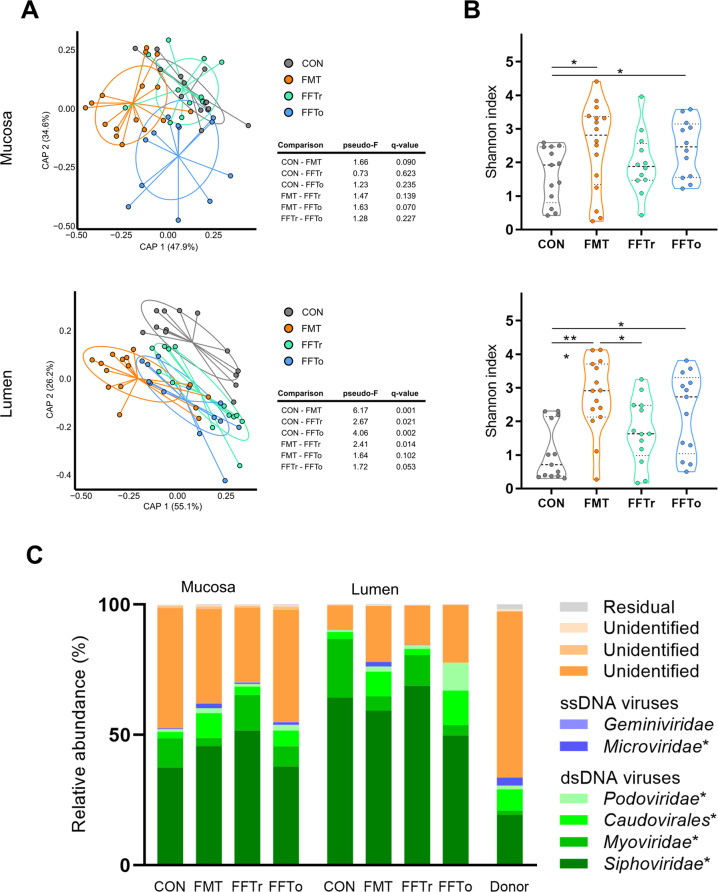
Viral and phage composition of gut mucosa and lumen. **A** Principal component analysis plots visualizing beta diversity based on constrained Bray–Curtis dissimilarity. FDR-adjusted probability levels of pairwise comparisons are reported in adjacent tables. **B** Shannon index as a measure of alpha diversity. **C** Mean relative viral abundance in gut mucosa and lumen summarized at family level. Taxa with more than 0.5% relative abundance across groups were included. Viral composition of donor fecal material is included as reference. Viral taxa marked with an asterisk are bacteriophages. Asterisk and double asterisks denote statistical probability levels below 0.05 or 0.001, respectively.

The luminal virome of FMT animals differed from all remaining groups, and both FFT groups differed from CON, whereas the effect of FFT administration route was only borderline significant (Fig. 4A). Interestingly, as opposed to bacteria, the Shannon index of mucosa-associated viruses increased following FMT and FFTo, but not FFTr relative to CON (both *p* *<* 0.05, Fig. 4B). The same pattern was observed for viral Shannon index in the luminal compartment. No correlation between mucosal viral Shannon index and small intestinal NEC severity was observed (data not shown). The majority of identifiable viruses across the groups were prokaryotic viruses (phages) primarily belonging to the order *Caudovirales* (Fig. 4C), although eukaryotic viruses were also identified but in low relative abundance (less than 1%). Notably, while FMT led to mucosal and luminal enrichment of eukaryotic virus families (e.g. *Herpesviridae*) relative to CON, this was not the case for FFT treatment (Supplementary Fig. S5). The number of relative differentially abundant viral OTUs was again highest in the gut lumen of the FMT recipients, but for the mucosa-associated virome, oral but not rectal FFT administration changed as many viral OTU relative abundances as FMT. Remarkably, several members of the phage family *Microviridae*, which naturally prey on enterobacteria, were increased in relative abundance only in the mucosa of the orally administered FFT group (Supplementary Fig. S5).

### Host gut mucosal transcriptome profiling

**Fig. 5:**
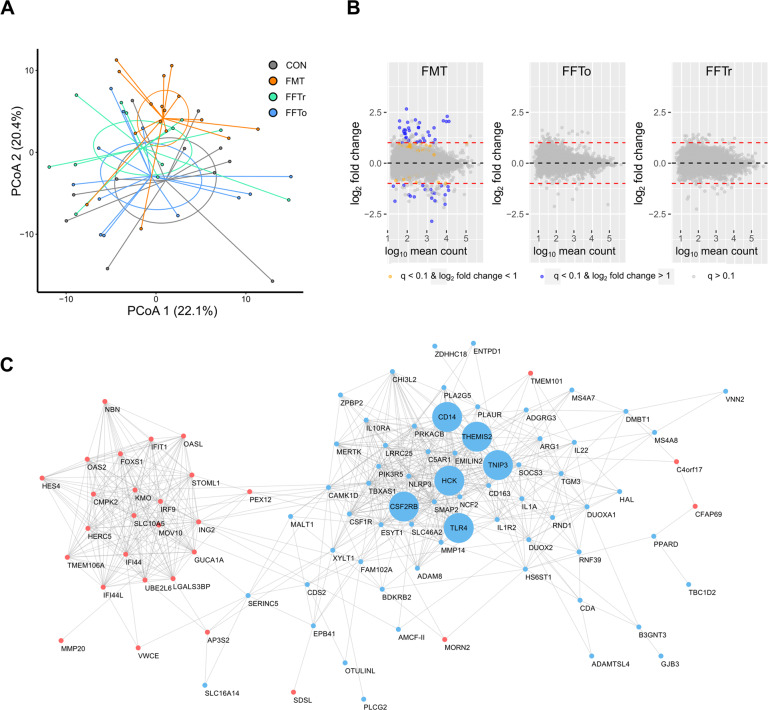
Host gut mucosal transcriptome profiling. **A** Principal component analysis plot of global gene expression differences based on RNA Seq. data. **B** Volcano plots of pairwise comparisons with the CON group, highlighting differentially expressed genes based on false discovery rate-adjusted statistics and fold-change criteria. **C** Gene interaction network showing FMT-upregulated genes in blue and FMT-downregulated genes in red. The genes with the highest number of interactions are highlighted with larger nodes.

To gain insight into the host mucosal response to treatment, we then performed RNA-Seq on ileal mucosa samples. Initially, a plotting of principal components showed that the FMT group separated from the remaining groups along the second principal component (Fig. 5A). Notably, for the two FFT groups we found no statistically significant differentially expressed genes relative to CON (FDR adjusted *p* *<* 0.10), whereas FMT increased the expression of 86 genes and decreased the expression of 41 genes compared with CON (Fig. 5B). When applying fold-change criteria (log_2_ > 1), 29 and 16 known genes were up- and downregulated by FMT relative to CON, respectively (Supplementary Table S2). A network analysis of differentially expressed genes identified lipopolysaccharide (LPS) response genes (e.g. *TLR4, CD14*, *THEMIS2*, *TNIP3*) as key genes in the FMT-enriched network, whereas several interferon-induced genes such as *IFIT1* and *OASL* were downregulated by FMT (Fig. 5C). Indeed, functional annotation of differentially expressed genes in FMT vs. CON mucosa showed that the most significantly affected pathways were related to immune activation and host defense mechanisms. Interestingly, FMT upregulated genes involved in bacterial response pathways and downregulated genes related with viral response (Supplementary Table S3).

### Systemic immune cell characterization

Finally, we measured the levels of basic immune cell subtypes in the bloodstream both shortly after intervention and at euthanasia to investigate any induction of systemic immunity. On day 3, i.e. shortly after the final treatment administration, we found marginally increased neutrophil counts in all intervention groups relative to CON (all *p* *<* 0.05, Supplementary Fig. S6), whereas monocyte and total lymphocyte levels were not affected. However, the helper T cell (CD4^+^CD8^−^) and naive T cell fraction (CD4^−^CD8^−^) were increased and decreased, respectively in FMT relative to FFTr (both *p* *<* 0.05). Two days later, these differences had all disappeared.

## Discussion

Despite being recognized for decades, NEC remains a clinical challenge today. Currently, disease prophylaxis is limited to the use of breastfeeding as well as probiotics, which lack standard recommendations and is subject to scrutiny [18]. The treatment of suspected NEC consists of enteral feeding discontinuation, enteral or parenteral antibiotics and symptomatic medical treatment. Still, almost 10% of extremely preterm infants develop NEC, and among these, the risk of death or disability is high [19]. Moreover, the widespread use of antibiotics in preterm infants due to suspected infection is related with an increase in bacterial antibiotics resistance [20], while animal experiments indicate that neonatal antibiotics perturb immune development in a microbiota-dependent manner and increase the risk of secondary infections [21, 22]. Collectively, there is a need for more effective therapeutic options with less collateral impact.

Here we aimed to test the NEC-preventive effect of FFT with an additional focus on preclinical safety. Using cesarean-delivered preterm pigs, the most clinically relevant animal model of NEC, we showed (1) a proof of principle for FFT treatment with a slight advantage of oral vs. rectal administration route (2) an inconspicuous FFT safety profile, whereas cognate FMT treatment was associated with a range of adverse effects. This study is the first to describe the use of FFT as a means to protect against NEC, and among the first to describe the therapeutic potential of FFT altogether. A small case report described the successful use of FFT in recurrent *Clostridioides difficile*-infected patients, the only patient group where FMT is routinely used. Besides ensuring clinical remission, FFT changed the gut bacterial and viral composition within each patient [11]. Recently, FFT from lean mice donors was shown to change the bacterial and viral gut microbiota, in turn reducing weight gain and improving glucose tolerance in diet-induced obese mice [12]. These studies both administered FFT in the proximal gut, while we additionally assessed the effects of gastric vs. rectal administration, and although only modestly different, the effect of gastric administration was superior in reducing pathological severity.

In our previous FMT experiment, combined gastric and rectal administration increased sepsis-related mortality [8]. We later found that antibiotic treatment prior to rectal FMT augmented NEC susceptibility [23]. In the current experiment, no adverse effects were observed during and following FFT treatment irrespective of administration route, while rectal FMT failed to prevent NEC, reduced total body growth and was detrimental to small intestinal structure and function. This is in contrast to our previous study investigating rectally administered FMT, where the clinical data indicated that the procedure was safe [8]. Importantly, the exact same donor material was used in the two experiments, and study designs including pathological assessments were identical. Hence, the inconsistency in the clinical response to rectal FMT cannot be explained using available data.

Phage density is increased in the mucus layer relative to the lumen [24], which inspired us to investigate effects of treatment on the mucosa-associated microbiota. Whereas all groups contained Enterobacteriaceae as part of the luminal microbiota, we found that CON and to a lesser extent FMT animals relative to FFTo had an increased relative abundance of this NEC-associated bacterial family in their mucosa [4, 6, 7, 25]. Moreover, the diversity of mucosa-associated bacteria correlated positively with NEC severity. As oro-gastric FFT increased mucosa-associated viral diversity, a potential mechanism of action for FFT might be a particular enrichment of phages in the mucus layer, which in turn reduces the relative abundance of certain bacteria e.g. Enterobacteriaceae in close proximity to the mucosa. Interestingly, we found an increased relative abundance of *Microviridae* in the mucosa of animals given oral FFT. This is in accordance with a recent study in human patients successfully treated with FMT to cure a *C. difficile* infection, wherein opposite inverse correlations between Proteobacteria and *Microviridae* were found before and after FMT treatment [26]. Of note, we cannot rule out the possibility that other substances than phages in the fecal filtrate such as microbial metabolites or secreted proteins might be responsible for the observed effects. Likewise, based on a recent metagenomic assessment of human fecal filtrate produced in identical fashion [27], we expect a minor fraction of small bacteria to be present in the filtrate but deem this of negligible clinical importance.

A peculiar finding concerned the colonization of recipients with *Streptococcus* and *Lactobacillus*, which together constituted a major fraction of the donor microbiota. While these genera were not detected in the gut of control animals, *Streptococcus* was the second most abundant in FFT animals, whereas *Lactobacillus* was hardly detectable. Contrarily, *Lactobacillus* was the second most abundant genus in FMT animals, a confirmation of previous observations [8], while *Streptococcus* relative abundance was reduced. Whether this dichotomy is of any clinical relevance, remains to be seen.

A major concern and potential obstacle for the use of FFT in preterm infants is the risk of transferring eukaryotic viruses capable of infecting human cells from an older donor individual into a compromised recipient [28]. In this study, phages, particularly those belonging to the order *Caudovirales*, were dominating across groups and anatomical niches. The second most abundant virus could not be identified, but as the five other most abundant viruses (totaling 98% relative abundance) were all phages, and since phage database coverage is much lower than for eukaryotic viruses, likely it is an uncharacterized phage. Indeed, *Caudovirales* is the dominating order of phages in infants as well [9, 10], and as importantly, relatively few eukaryotic viruses inhabit the newborn as well as the 1-month-old infant gut, whereas the relative abundance increases at 4 months [10]. Regardless, we found that FMT but not FFT increased the relative abundance of a number of clinically relevant mammalian viruses e.g. Herpesviridae. Either the filtering process inhibits a fraction of the largest virus particles from entering the FFT solution, or in vivo conditions following FFT prevent these viruses from infecting host cells. In any case, this aspect is important for FFT clinical feasibility.

To increase the understanding of how gut microbiota-directed therapies may act or interact to prevent intestinal pathology, we performed a global gene expression analysis of the host gut mucosa. This analysis was performed in anatomically standardized tissue specimens, which were largely devoid of severe pathology. Hence, the results should be interpreted as isolated treatment effects with minimal bias due to pathological state. Strikingly, no gene expression levels were significantly affected by FFT relative to CON, which indicates that no particular interaction occurs between administered viruses (or small molecules) and host immune cells, implying that FFT exerts its effect on bacteria only. Direct interaction between phages and host via TLR9 signaling has been demonstrated under germ-free conditions [29], but under physiological conditions, this effect may be of negligible importance. On the other hand, FMT treatment regulated mucosal gene expression levels towards increased immune response to bacteria and concomitant reduction in host response to viruses. Specifically, a series of genes involved in the LPS signaling pathway were upregulated, implicating LPS-producing Enterobacteriaceae in the FMT-related side effects. Furthermore, FMT led to a transient induction of circulating helper T cells shortly after administration. From a safety perspective, the lack of collateral effects on host immunity favors the use of FFT over FMT. However, in more robust individuals, the induction of mucosal immunity by FMT might be beneficial.

Collectively, we have uncovered the potential of FFT as a safe and efficient means to reduce NEC in preterm neonatal pigs. As the FFT concept has been demonstrated in the porcine species only, and using just a single batch of donor fecal material, a generalized conclusion across species and individual donors in terms of safety and efficacy cannot be made yet. Regardless, in light of these findings and the unique potential, we encourage FFT safety and feasibility testing in preterm infants using a rigid safety paradigm. Donor and stool should be subject to an extensive screening procedure, equivalent to existing pediatric FMT procedures [30], and bacteria removed using validated techniques [13].

## Supplementary information

Supplementary methods

Supplementary figures and tables

## Data Availability

All generated nucleotide sequencing reads will be made available through publicly accessible data repositories. Mucosa RNAseq reads have been uploaded to Gene Expression Omnibus (accession number GSE158878), and 16S rDNA gene amplicon and meta-virome reads are uploaded to the NCBI Sequence Read Archive (accession number PRJNA736598) and will be released upon manuscript publication.
