# Supplementary material for: Fecal filtrate transplantation protects against necrotizing enterocolitis
Source: ISME J. 2021 Sep 22;16(3):686–94. doi: 10.1038/s41396-021-01107-5 (PMC8857206; doi:10.1038/s41396-021-01107-5)
Supplement: Supplementary file 1 — Supplementary methods [file 41396_2021_1107_MOESM1_ESM.docx]

# SUPPLEMENTARY METHODS

**16S rRNA gene amplicon sequencing bioinformatics workflow**

The raw sequencing reads were merged and trimmed. Chimeras were removed and zero-radius Operational Taxonomic Units (zOTUs) constructed using UNOISE algorithm implemented in Vsearch[1–3]. The Greengenes (version 13.8) database was used as reference for annotation. QIIME2 was used to process the forward analysis. Rare zOTUs with frequency below 0.1% of the minimal sample depth were filtered and removed, and based on rarefaction curve the zOTU table was rarified to adequate sample depth (9000 counts) for alpha and beta diversity calculations. Principal coordinate analysis (PCoA) was conducted on unweighted UniFrac dissimilarity metrics, a PERMANOVA test with FDR correction was performed to detect pairwise group differences. Wilcoxon rank-sum test was used to analyze pairwise group differences in Shannon index. Differences in relative abundance at single zOTU level between control and treatment groups was analyzed by DESeq2[4].

**Virome sequencing bioinformatics workflow**

The raw reads were trimmed from adaptors and barcodes and the high quality sequences (>95% quality) using Trimmomatic v0.35[5], with a minimum size of 50nt were retained for further analysis. High quality reads were de-replicated and checked for the presence of Phix179 using USEARCH v10[6]. Virus-like particle-derived DNA sequences were subjected to within-sample *de-novo* assembly-only using Spades v3.13.1[7] and the contigs with a minimum length of 2,000 nt, were retained. Contigs generated from all samples were pooled and de-replicated by multiple blasting, thus removing those contained in over 90% of the length of another (90% similarity). To check the presence of non-viral DNA contigs, de-replicated contigs were subjected to the following pipeline: (i) contigs classified as of human origin by *k-*mer profiles (Kraken2[8], --confidence 0.1) and (ii) plant genomes (NCBI) *k-*mers profiles (Kraken2, --confidence 0.3) were discarded. Contigs that either aligned (blastn, minimum alignment score of 30% of the target or query) to the (iii) cultured and uncultured collection of viral sequences hosted at the IMG/VR v2.0 database[9], (iv) the human gut virome (GVD) database[10], (v) selected as Category 1-3 viruses by VirSorter[11], (vi) showed a *p-*value < 0.05 within VirFinder[12], (vii) contained at least 2 different viral orthologous proteins in different locations of their genomes, (viii) or classified as viral given their *k-*mer signature (Kraken2) based on the NCBI viral genomes database, were retained for downstream analyses. Contigs that did not meet the above criteria were searched by their *k-*mer signatures against the (ix) NCBI plamids database (Kraken2) and (x) NCBI bacterial genomes (Kraken2, --confidence 0.01), the contigs that remained unclassified were also retained from downstream analyses. Following assembly and quality control, reads from all samples were mapped against the remaining (high-quality) contigs using the Subread aligner[13] and a contingency table of reads per kilobase of contig sequence per million reads sample was generated, here defined as vOTU table (viral operational taxonomic unit). Taxonomic annotation of contigs was determined as follows: ORF calling and gene predictions on HQ-contigs were performed with Prodigal[14], the predicted proteins were blasted (blastp) against NCBI NR viral protein database. Using Basic Sequence Taxonomy Annotation tool[15], the Lowest Common Ancestor (LCA) for every contig was estimated based on: percentage of hits of LCA of 60, minimum identity of 0.4, minimum alignment of 0.4 and a minimum number of hits for LCA of 10. Analysis of viral community α- and β-diversity were performed using packages Phyloseq v1.30.0[16] and Vegan 2.5-6[17] in R statistical software. For α-diversity analysis, Shannon index was calculated. Bray-Curtis distance metrics were calculated for β-diversity analysis. Constrained ordination was done using distance-based redundancy analysis (dbRDA, ‘capscale)’ function in Vegan). Pairwise group differences were assessed with PERMANOVA. Wilcoxon rank-sum test was used to analyze pairwise group differences in Shannon index, while pair-wise group comparisons in relative abundance of specific taxa was analyzed by DESeq2[4].

**RNA Seq bioinformatics workflow**

Quality and adapter trimming of raw reads was performed using Trim Galore (Babraham Bioinformatics). Alignment against the porcine reference genome Sscrofa11.1 was performed with the RNA-Seq aligner Tophat[18]. Gene counts were obtained with HTSeq-count[19], using gene annotations from the Ensembl genome assembly (v91). Statistical analysis of differential gene expression between groups was performed by DESeq2[4] in R using an FDR adjusted cutoff of p<0.10. Putative co-expression gene network was conducted using pairwise spearman correlation to determine possible gene pairs, where correlation with absolute spearman rho >0.6 as well as FDR <0.05 was considered statistically significant. STRING database was used for functional enrichment analysis. Biological processes and KEGG pathways with FDR <0.05 were considered statistically significant.

**SUPPLEMENTARY REFERENCES**

1 Edgar RC. UNOISE2: improved error-correction for Illumina 16S and ITS amplicon sequencing. *bioRxiv* 2016;:10.1101/081257. doi:10.1101/081257

2 Rognes T, Flouri T, Nichols B, *et al.* VSEARCH: A versatile open source tool for metagenomics. *PeerJ* 2016;**4**:e2584. doi:10.7717/peerj.2584

3 Edgar RC. UCHIME2: improved chimera prediction for amplicon sequencing. *bioRxiv* 2016;:10.1101/074252. doi:10.1101/074252

4 Love MI, Huber W, Anders S. Moderated estimation of fold change and dispersion for RNA-seq data with DESeq2. *Genome Biol* 2014;**15**:550. doi:10.1186/s13059-014-0550-8

5 Bolger AM, Lohse M, Usadel B. Trimmomatic: A flexible trimmer for Illumina sequence data. *Bioinformatics* 2014;**30**:2114–20. doi:10.1093/bioinformatics/btu170

6 Edgar RC. Search and clustering orders of magnitude faster than BLAST. *Bioinformatics* 2010;**26**:2460–1. doi:10.1093/bioinformatics/btq461

7 Bankevich A, Nurk S, Antipov D, *et al.* SPAdes: A new genome assembly algorithm and its applications to single-cell sequencing. *J Comput Biol* 2012;**19**:455–77. doi:10.1089/cmb.2012.0021

8 Wood DE, Lu J, Langmead B. Improved metagenomic analysis with Kraken 2. *Genome Biol* 2019;**20**:257. doi:10.1186/s13059-019-1891-0

9 Paez-Espino D, Roux S, Chen IMA, *et al.* IMG/VR v.2.0: An integrated data management and analysis system for cultivated and environmental viral genomes. *Nucleic Acids Res* 2019;**47**:D678–86. doi:10.1093/nar/gky1127

10 Gregory A, Zablocki O, Howell A, *et al.* The human gut virome database. *bioRxiv* 2019;:655910. doi:10.1101/655910

11 Roux S, Enault F, Hurwitz BL, *et al.* VirSorter: Mining viral signal from microbial genomic data. *PeerJ* 2015;**2015**:e985. doi:10.7717/peerj.985

12 Ren J, Ahlgren NA, Lu YY, *et al.* VirFinder: a novel k-mer based tool for identifying viral sequences from assembled metagenomic data. *Microbiome* 2017;**5**:69. doi:10.1186/s40168-017-0283-5

13 Liao Y, Smyth GK, Shi W. The Subread aligner: Fast, accurate and scalable read mapping by seed-and-vote. *Nucleic Acids Res* 2013;**41**:e108. doi:10.1093/nar/gkt214

14 Hyatt D, Chen GL, LoCascio PF, *et al.* Prodigal: Prokaryotic gene recognition and translation initiation site identification. *BMC Bioinformatics* 2010;**11**:119. doi:10.1186/1471-2105-11-119

15 Kahlke T, Ralph PJ. BASTA – Taxonomic classification of sequences and sequence bins using last common ancestor estimations. *Methods Ecol Evol* 2019;**10**:100–3. doi:10.1111/2041-210X.13095

16 McMurdie PJ, Holmes S. phyloseq: An R Package for Reproducible Interactive Analysis and Graphics of Microbiome Census Data. *PLoS One* 2013;**8**:e61217. doi:10.1371/journal.pone.0061217

17 Dixon P. VEGAN, a package of R functions for community ecology. J. Veg. Sci. 2003;**14**:927–30. doi:10.1111/j.1654-1103.2003.tb02228.x

18 Trapnell C, Pachter L, Salzberg SL. TopHat: Discovering splice junctions with RNA-Seq. *Bioinformatics* 2009;**25**:1105–11. doi:10.1093/bioinformatics/btp120

19 Anders S, Pyl PT, Huber W. HTSeq-A Python framework to work with high-throughput sequencing data. *Bioinformatics* 2015;**31**:166–169. doi:10.1093/bioinformatics/btu638
