# Supplementary material for: Fecal filtrate transplantation protects against necrotizing enterocolitis
Source: ISME J. 2021 Sep 22;16(3):686–94. doi: 10.1038/s41396-021-01107-5 (PMC8857206; doi:10.1038/s41396-021-01107-5)
Supplement: Supplementary file 2 — Supplementary figures and tables [file 41396_2021_1107_MOESM2_ESM.docx]

**SUPPLEMENTARY MATERIAL**

**
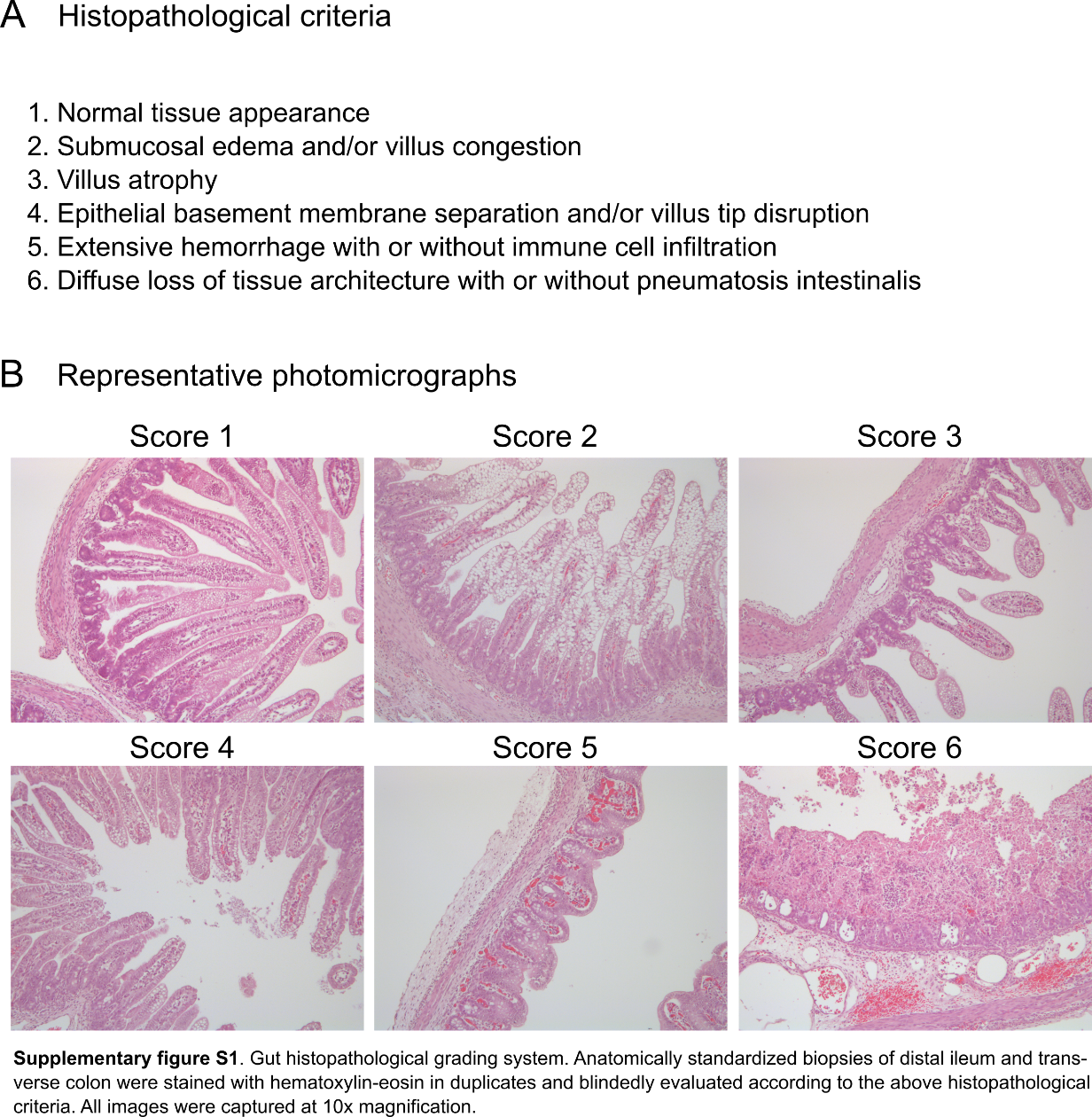
**

**Supplementary figure S1. Presentation of gut histopathological grading system**. A. Definition of the six grades of increasing histopathological severity. B. Representative micrographs illustrating each severity grade.


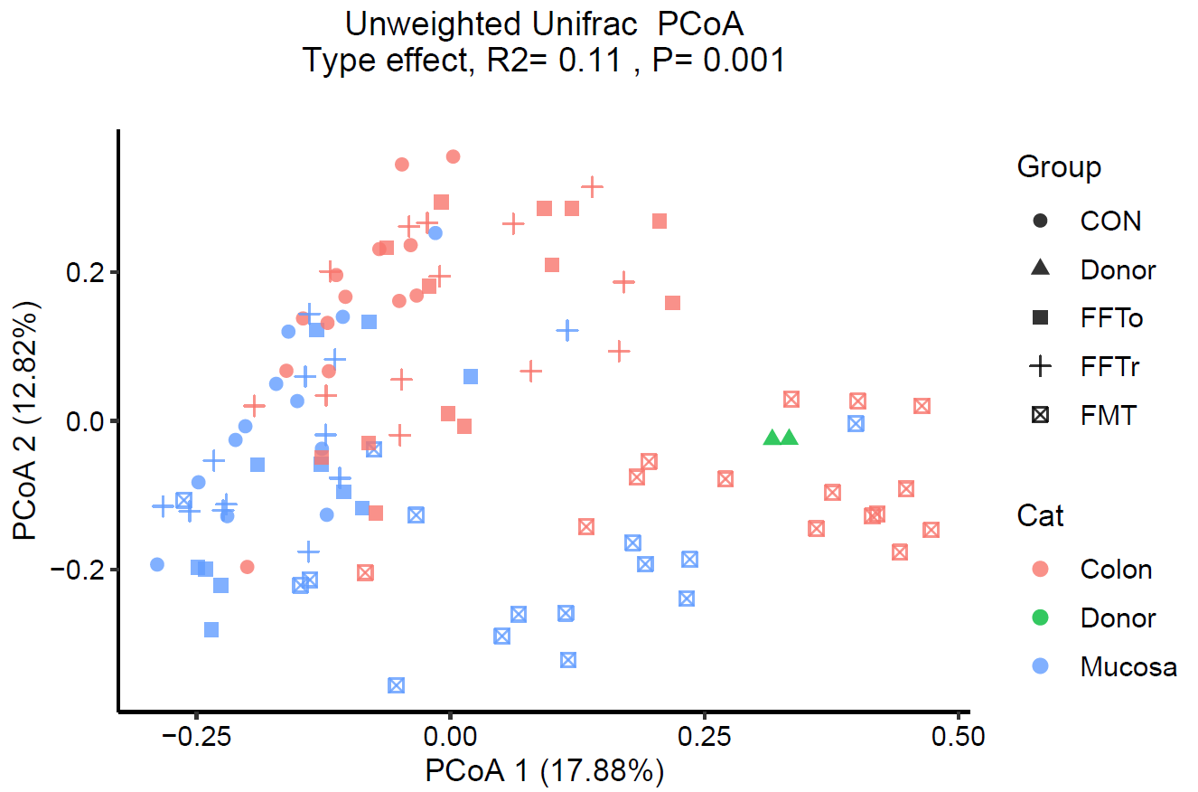


**Supplementary figure S2. Bacterial composition in different gut niches**. Comparison of bacterial composition in the gut mucosal and luminal niche presented as principal component analysis plot based on unweighted UniFrac dissimilarity.


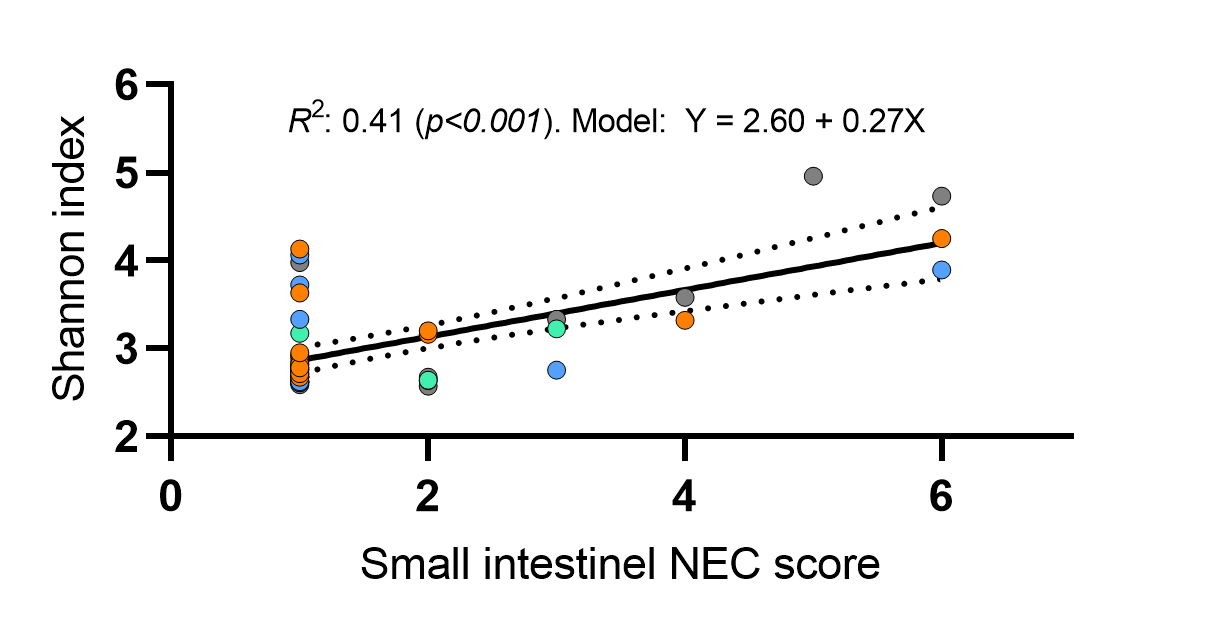


**Supplementary figure S3. Association between macroscopic NEC severity of the small intestine and mucosa bacterial diversity as assessed by Shannon index**.

**
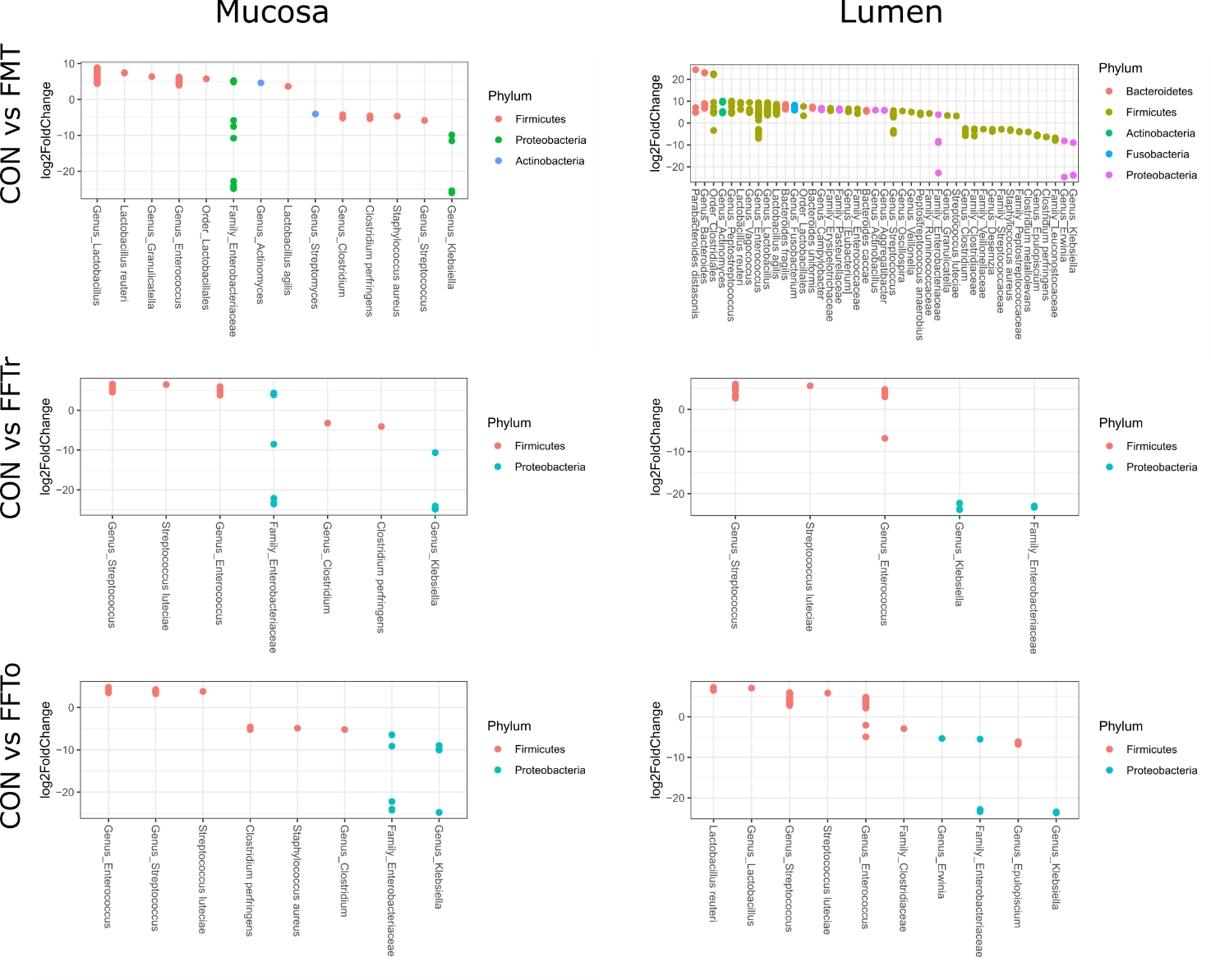
Supplementary figure S4. Relative differentially abundant single bacterial OTUs in mucosa and lumen.** Single dots denote bacterial OTUs with significantly different relative abundance compared with controls (CON) based on DESeq2 analysis with unadjusted probability levels below 0.01 considered significant. OTUs are sorted on the x-axis from left to right according to the log_2_ fold difference in relative abundance and colored based on bacterial taxonomy at phylum level.

**
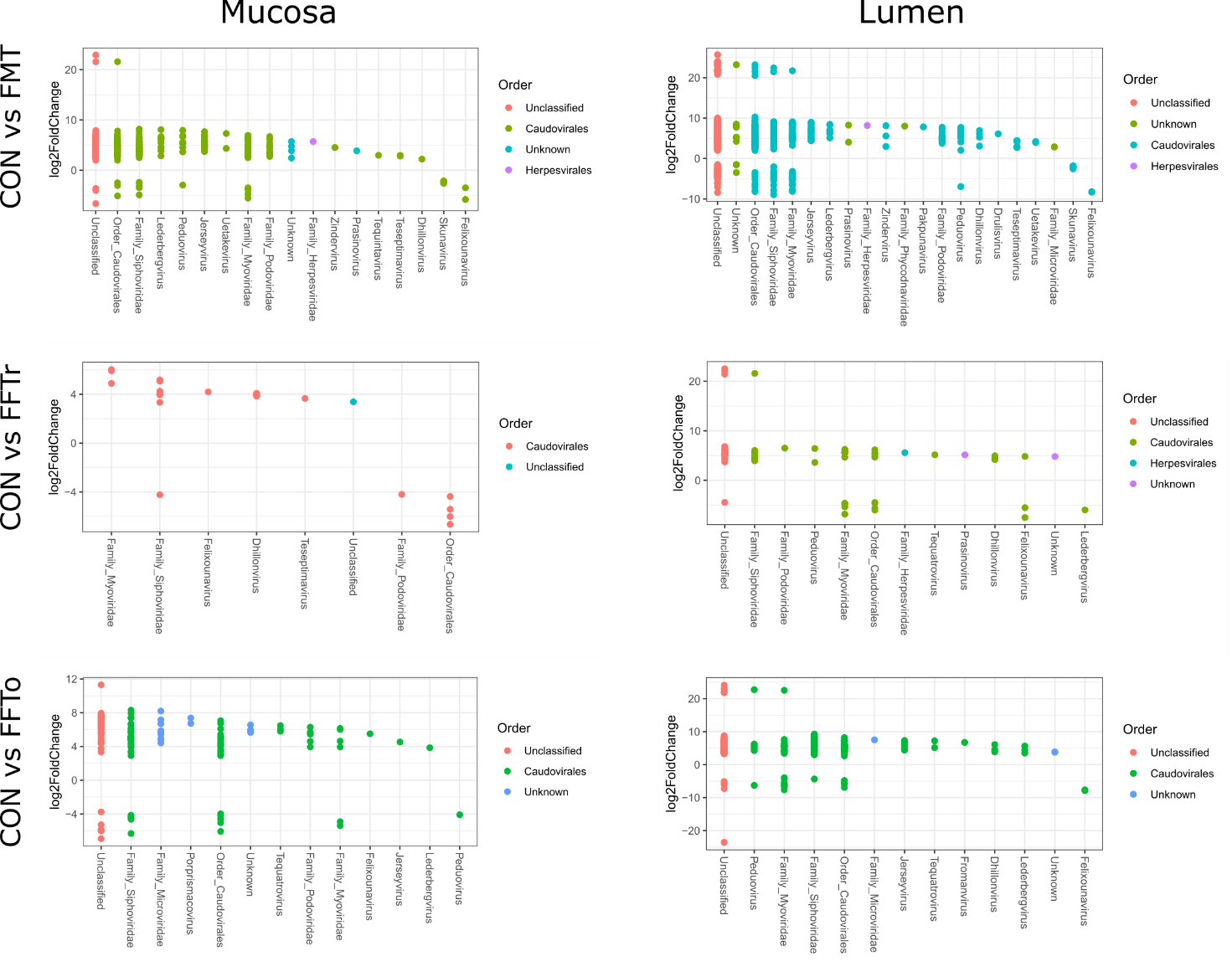
Supplementary figure S5. Relative differentially abundant single viral OTUs in mucosa and lumen.** Single dots denote viral OTUs with significantly different relative abundance compared with controls (CON) based on DESeq2 analysis with unadjusted probability levels below 0.01 considered significant. OTUs are sorted on the x-axis from left to right according to the log_2_ fold difference in relative abundance and colored based on viral taxonomy at order level. Unclassified viral OTUs represent taxa unclassified by the viral databases, and Unknown viral OTUs represent taxa identified as viruses in the database but without exact taxonomic classification.


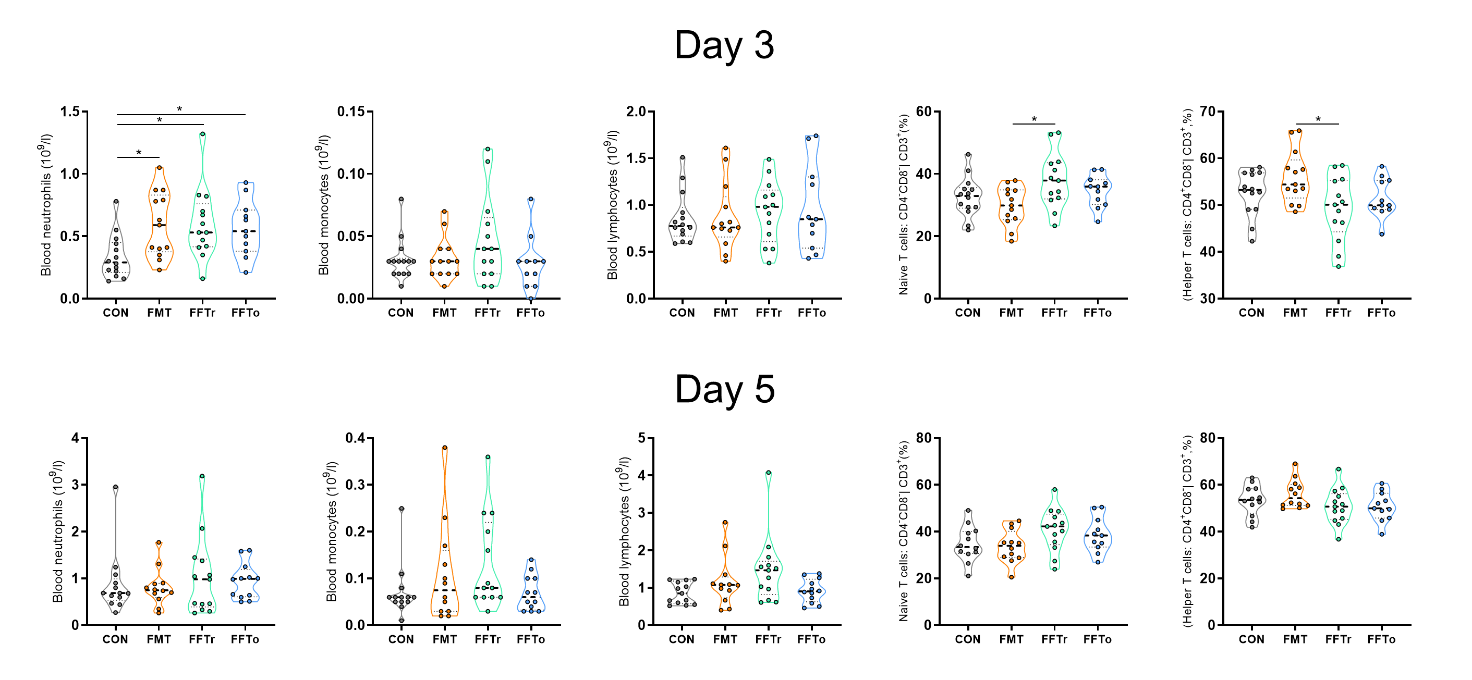


**Supplementary figure S6. Systemic immune cell characterization following FMT or FFT treatment.** Blood neutrophils, monocytes and total lymphocytes as well as helper T cell and naïve T cell fractions were measured on day 3 shortly after treatments and again on day 5. * denotes a statistical probability level below 0.05.

**Supplementary table S1.** Infant formula composition

| **Constituent** | **Infant formula** |
| --- | --- |
| Fantomalt | 40 g/L |
| Seravit | 12 g/L |
| Miprodan 40 | 15 g/L |
| Lacprodan DI-9224 | 60 g/L |
| Liquigen | 75 g/L |
| Calogen | 20 g/L |

**Supplementary table S2**. Mucosal RNA seq. differentially expressed genes.

| **Gene** | **CON** | **FMT** | **FFTr** | **FFTo** | **log2FoldChange (FMT/CON)** | **FDR adj. P-value** |
| --- | --- | --- | --- | --- | --- | --- |
| ARG1 | 27 | 181 | 67 | 17 | 2,66 | 0,05 |
| TGM3 | 3859 | 18896 | 8240 | 4811 | 2,29 | 0,02 |
| MMP9 | 99 | 426 | 218 | 122 | 2,12 | 0,04 |
| MT1A | 17 | 83 | 17 | 12 | 2,07 | 0,08 |
| IL22 | 37 | 166 | 77 | 70 | 2,00 | 0,05 |
| DUOXA1 | 150 | 665 | 367 | 217 | 1,98 | 0,06 |
| CDA | 158 | 544 | 398 | 184 | 1,75 | 0,04 |
| MMP13 | 192 | 607 | 210 | 227 | 1,75 | 0,05 |
| PLA2G5 | 18 | 64 | 32 | 21 | 1,74 | 0,02 |
| DUOX2 | 1111 | 3695 | 2009 | 937 | 1,73 | 0,07 |
| MS4A7 | 58 | 197 | 61 | 73 | 1,73 | 0,05 |
| VNN2 | 38 | 117 | 45 | 34 | 1,63 | 0,00 |
| GJB3 | 35 | 112 | 77 | 50 | 1,62 | 0,05 |
| IL1R2 | 64 | 192 | 100 | 72 | 1,60 | 0,03 |
| HAL | 39 | 108 | 41 | 33 | 1,55 | 0,07 |
| CD163 | 611 | 1836 | 759 | 671 | 1,53 | 0,04 |
| IL1A | 37 | 106 | 53 | 49 | 1,45 | 0,04 |
| CHI3L2 | 42 | 116 | 64 | 52 | 1,45 | 0,04 |
| RBP4 | 860 | 2018 | 819 | 810 | 1,36 | 0,07 |
| TNIP3 | 51 | 124 | 79 | 48 | 1,26 | 0,04 |
| AMCF-II | 361 | 872 | 556 | 439 | 1,23 | 0,04 |
| ADGRG3 | 49 | 114 | 50 | 61 | 1,21 | 0,03 |
| SOCS3 | 1585 | 3582 | 2134 | 1648 | 1,15 | 0,04 |
| RNF39 | 76 | 172 | 85 | 63 | 1,12 | 0,07 |
| MS4A8 | 467 | 1036 | 593 | 567 | 1,09 | 0,04 |
| PRSS22 | 281 | 598 | 363 | 319 | 1,07 | 0,07 |
| SLC46A2 | 56 | 117 | 114 | 87 | 1,06 | 0,01 |
| RND1 | 192 | 402 | 251 | 246 | 1,03 | 0,08 |
| CSF2RB | 1282 | 2679 | 1817 | 1445 | 1,00 | 0,03 |
| C4orf17 | 88 | 40 | 66 | 57 | -1,09 | 0,09 |
| HES4 | 972 | 368 | 387 | 599 | -1,11 | 0,08 |
| UBE2L6 | 6569 | 2951 | 4838 | 6180 | -1,12 | 0,07 |
| IRF9 | 25 | 12 | 15 | 21 | -1,14 | 0,04 |
| FOXS1 | 1992 | 775 | 1541 | 1680 | -1,30 | 0,05 |
| SDSL | 1197 | 482 | 427 | 951 | -1,30 | 0,04 |
| LGALS3BP | 16240 | 6317 | 10578 | 15171 | -1,34 | 0,09 |
| CMPK2 | 2555 | 901 | 1636 | 2330 | -1,46 | 0,09 |
| IFI44 | 3375 | 1159 | 2032 | 2903 | -1,48 | 0,10 |
| GUCA1A | 151 | 58 | 98 | 147 | -1,56 | 0,08 |
| OAS2 | 11636 | 3977 | 6700 | 9993 | -1,56 | 0,07 |
| MMP20 | 332 | 137 | 162 | 225 | -1,66 | 0,05 |
| HERC5 | 3820 | 1037 | 1895 | 3079 | -1,85 | 0,10 |
| VWCE | 40 | 8 | 11 | 32 | -1,87 | 0,03 |
| IFIT1 | 13239 | 3346 | 6123 | 9729 | -2,02 | 0,07 |
| OASL | 2565 | 611 | 1331 | 2280 | -2,86 | 0,08 |
| Differentially expressed genes in CON vs FMT with at log2 fold change > 1 and FDR adjusted test probability level < 0.10 summarized in descending order according to fold change level. | | | | | | |

**Supplementary table S3**. RNA seq. Gene ontology enrichment analysis.

| **Term ID** | **Term description** | **Gene count** | **Background** | **FDR adj. P-value** |
| --- | --- | --- | --- | --- |
| GO:0051707 | response to other organism | 25 | 835 | 3,99E-09 |
| GO:0006952 | defense response | 26 | 1234 | 6,30E-07 |
| GO:0002237 | response to molecule of bacterial origin | 14 | 317 | 9,75E-07 |
| GO:0031347 | regulation of defense response | 19 | 676 | 1,27E-06 |
| GO:0009617 | response to bacterium | 17 | 555 | 2,39E-06 |
| GO:0032496 | response to lipopolysaccharide | 13 | 298 | 2,76E-06 |
| GO:0009605 | response to external stimulus | 30 | 1857 | 4,93E-06 |
| GO:0051704 | multi-organism process | 33 | 2222 | 5,14E-06 |
| GO:0009615 | response to virus | 12 | 270 | 6,40E-06 |
| GO:0002252 | immune effector process | 20 | 927 | 1,61E-05 |
| GO:0006950 | response to stress | 40 | 3267 | 1,61E-05 |
| GO:0006954 | inflammatory response | 14 | 482 | 5,43E-05 |
| GO:0006955 | immune response | 25 | 1560 | 7,33E-05 |
| GO:0051607 | defense response to virus | 9 | 181 | 1,10E-04 |
| GO:0002682 | regulation of immune system process | 23 | 1391 | 1,20E-04 |
| GO:0002376 | immune system process | 31 | 2370 | 1,40E-04 |
| GO:0080134 | regulation of response to stress | 22 | 1299 | 1,40E-04 |
| GO:0071222 | cellular response to lipopolysaccharide | 8 | 146 | 1,80E-04 |
| GO:0032101 | regulation of response to external stimulus | 16 | 732 | 2,00E-04 |
| GO:0033993 | response to lipid | 17 | 825 | 2,00E-04 |
| Top 20 gene ontology terms in CON vs FMT summarized according to FDR adjusted test probability level. | | | | |
